# Supplementary material for: Impact of New York City’s 4-year multi-component natural experiment to improve elementary school physical education on student cardiorespiratory fitness
Source: BMC Public Health. 2024 Nov 14;24:3161. doi: 10.1186/s12889-024-20673-9 (PMC11562091; doi:10.1186/s12889-024-20673-9)

**Supplementary Materials - Parametric g-formula methods:**

Applying the parametric g-formula occurs in several steps. In Step 1, the observed outcome, “treatments”, and time-varying covariates are modeled, saving the estimated coefficients. Next, a Monte Carlo sample is drawn from the baseline data (school year 2014/15). A covariate history is generated for each school in the sample by predicting, for each subsequent school year, values for all time-varying covariates (including the sociodemographic composition of the school, implementation of PE Works components, and proportion of students meeting aerobic capacity HFZ standards) on the basis of prior values of these covariates and the coefficients estimated in step 1. This procedure is repeated under interventions on the PE Works components. For example, a counterfactual covariate history corresponding to “no PE Works components implemented” is generated by setting all PE Works components to zero at every year and using those values, the estimated coefficients from Step 1, and the other predicted covariates to predict each covariate under that scenario. We can then compare the expected proportion of students meeting fitness standards if all schools had received implementation of all PE Works components starting immediately in 2015/16 to what would have happened if PE Works had not been delivered at all. This procedure controls confounding by all time-varying covariates modeled as well as for any baseline variables included in the models.

In this application, the following variables were modeled for each school in each year: proportion of students meeting aerobic capacity HFZ standards (outcome); all four of the PE Works components (primary predictors); total school enrollment; proportion of students who qualified for FRPM; the proportion of White students; the total number of students tested; the number of PE teachers at the school; and the number of days of PE per week. Each of these depended on a relevant subset of baseline and time-varying covariates. The school-level proportion of students meeting fitness standards each year was modeled as a function of its value in the previous years; PE Works cohort; total number of PE teachers; number of audit conditions met; number of audit conditions changed after OSWP coaching; the number of total OSWP coaching interactions; Move-to-Improve All-Star status (both current and prior year); total school enrollment; proportion of students qualified for FRPM; proportion of White students; number of students tested; and the number of days of PE per week.

**Supplementary Figure 1. Adjusted school-level proportion of male students who met aerobic capacity Healthy Fitness Zone standards before (2014/15) and during PE Works (2015/16 – 2018/19) under observed and predicted PE Works conditions**


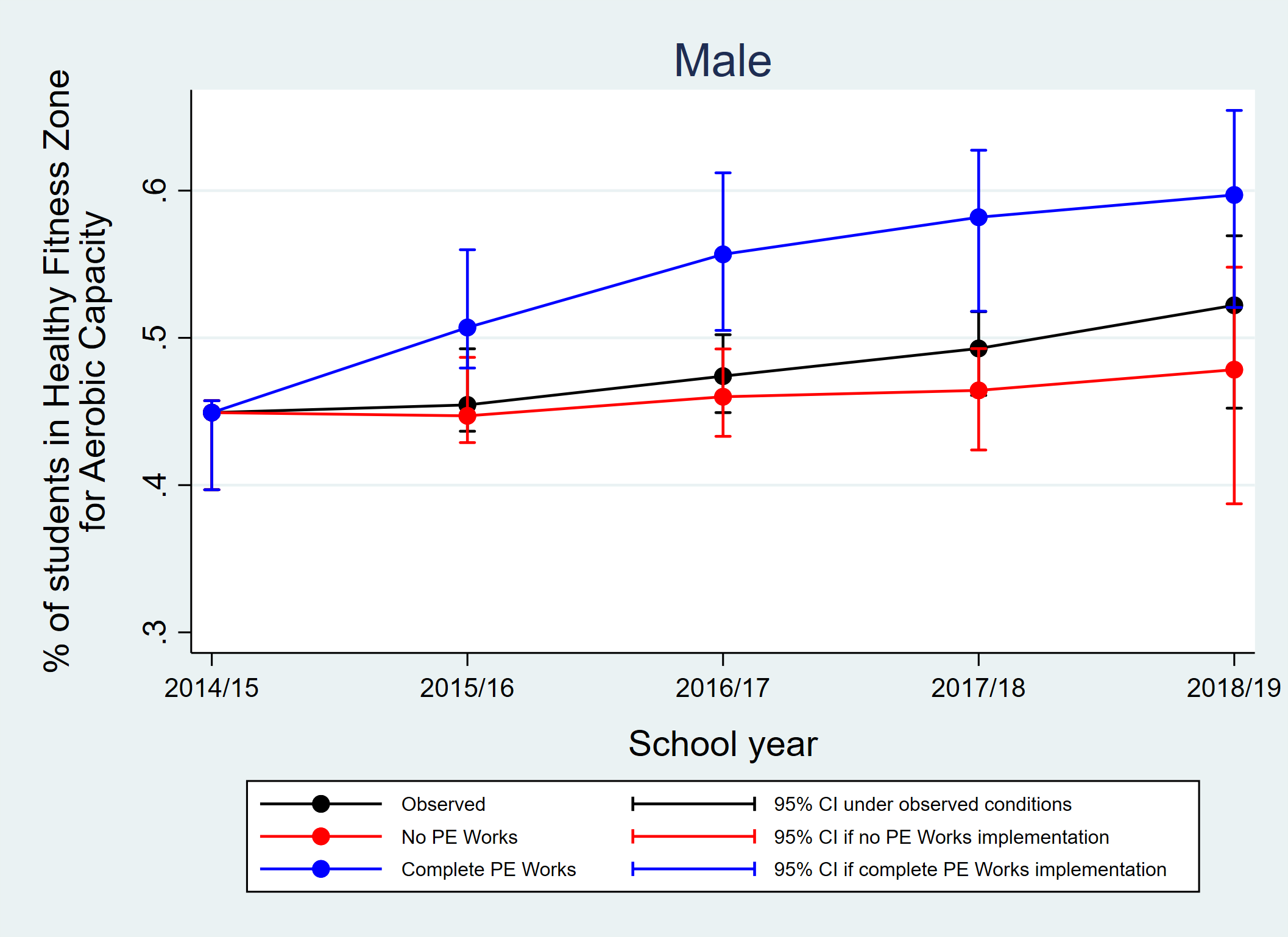


**Supplementary Figure 2. Adjusted school-level proportion of female students who met aerobic capacity Healthy Fitness Zone standards before (2014/15) and during PE Works (2015/16 – 2018/19) under observed and predicted PE Works conditions**


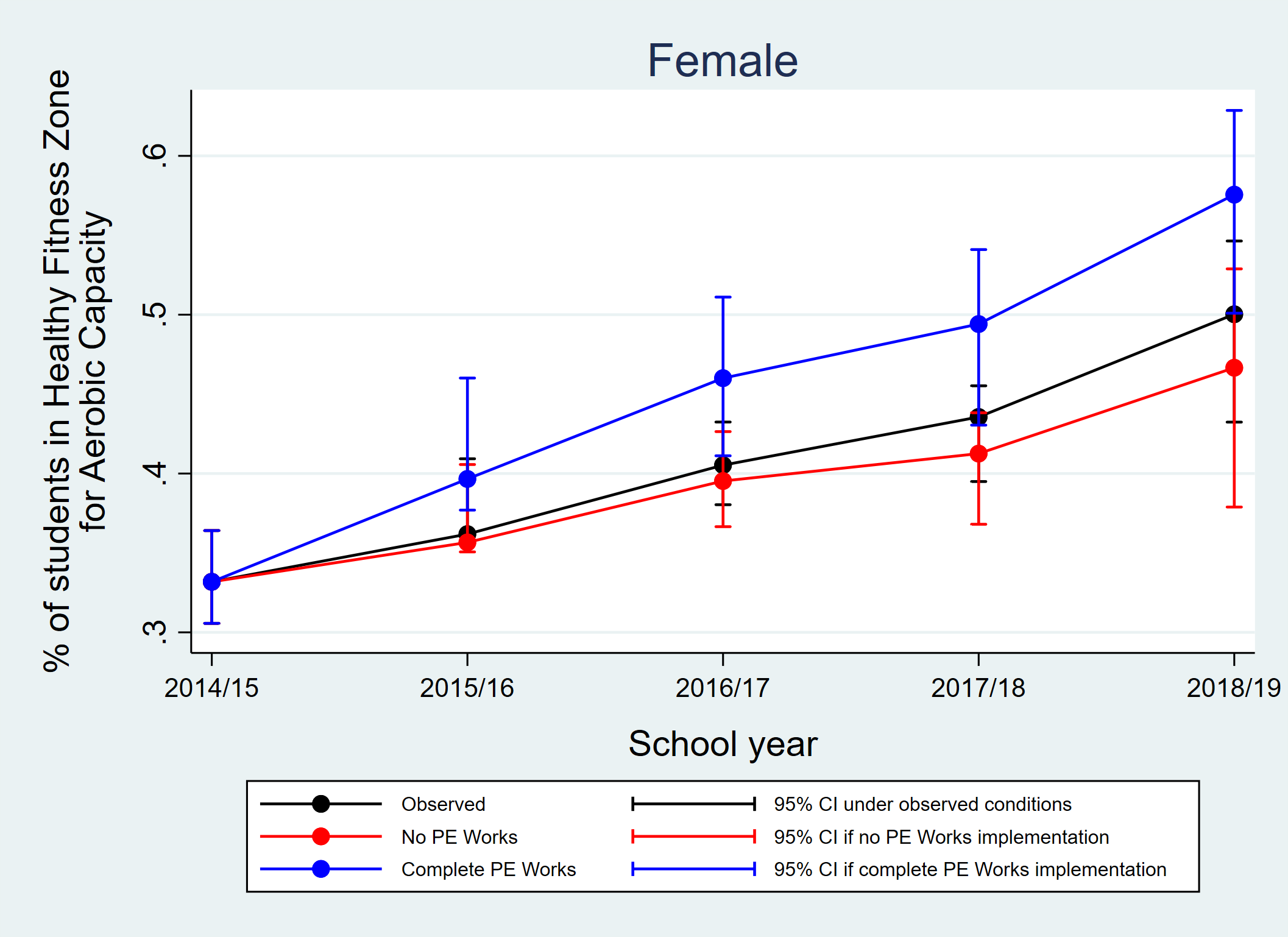


**Supplementary Figure 3. Adjusted school-level proportion of Asian American students who met aerobic capacity Healthy Fitness Zone standards before (2014/15) and during PE Works (2015/16 – 2018/19) under observed and predicted PE Works conditions**


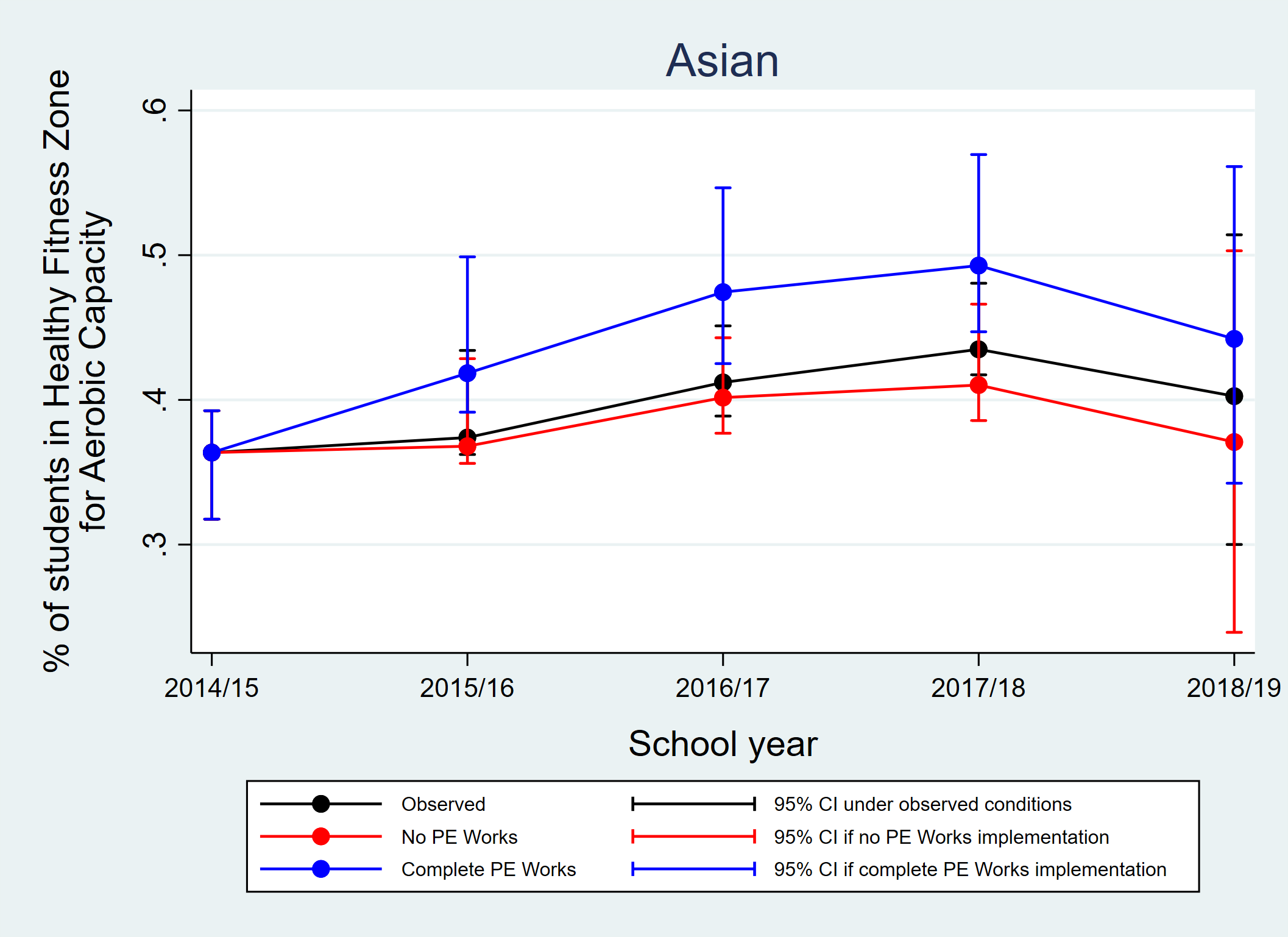


**Supplementary Figure 4. Adjusted school-level proportion of AfricanAmerican students who met aerobic capacity Healthy Fitness Zone standards before (2014/15) and during PE Works (2015/16 – 2018/19) under observed and predicted PE Works conditions**


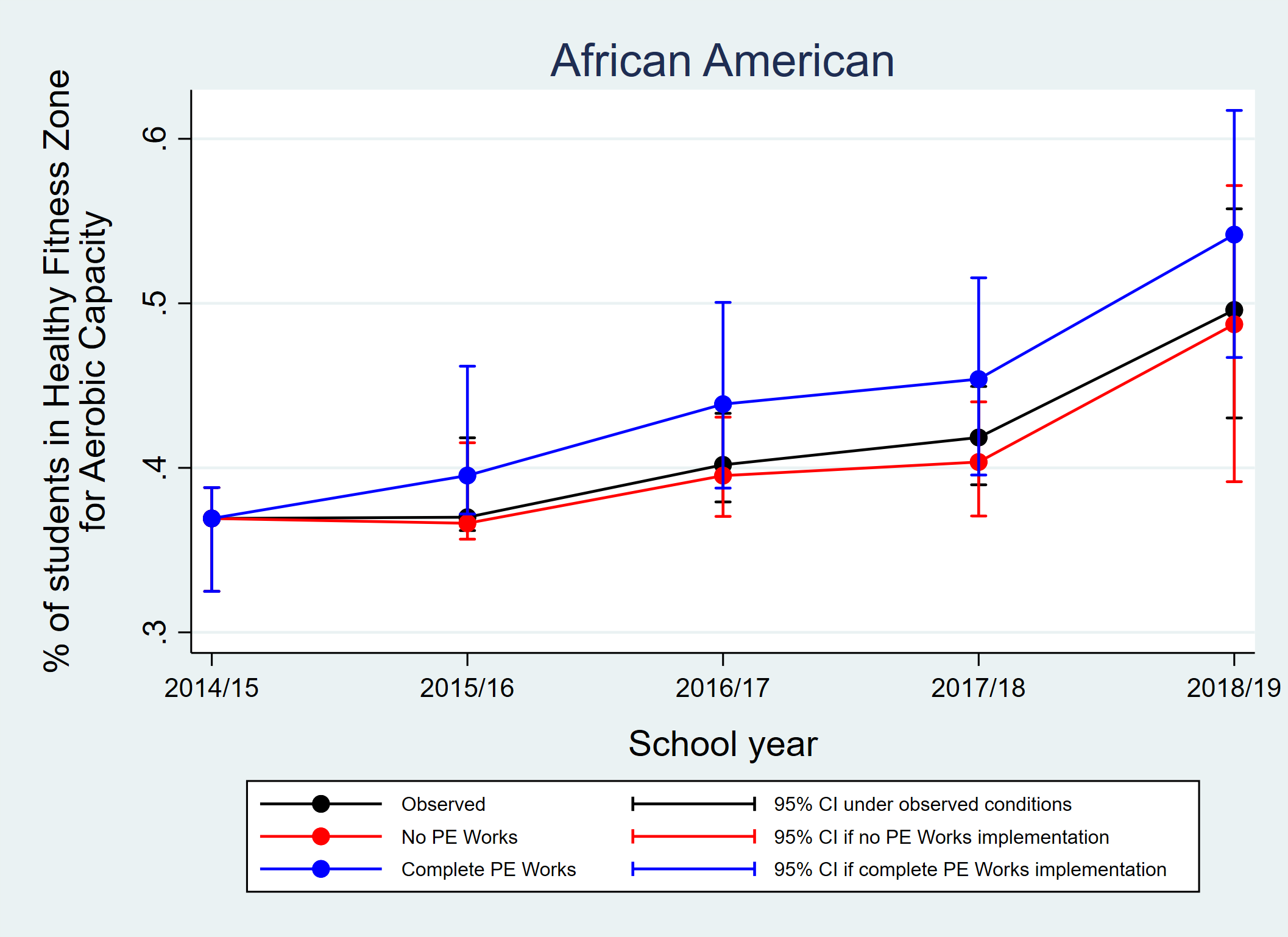


**Supplementary Figure 5. Adjusted school-level proportion of Hispanic/Latino students who met aerobic capacity Healthy Fitness Zone standards before (2014/15) and during PE Works (2015/16 – 2018/19) under observed and predicted PE Works conditions**


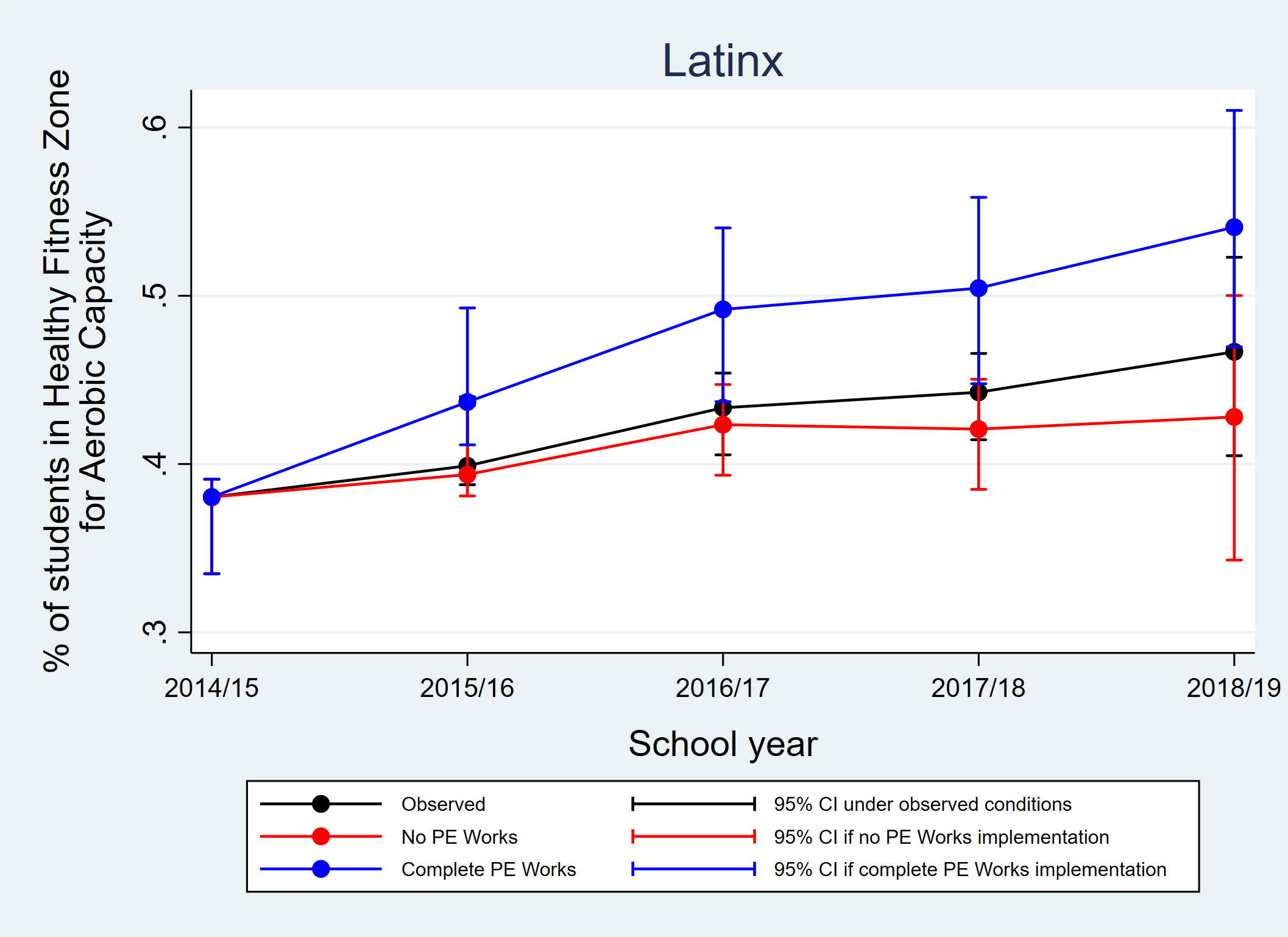


**Supplementary Figure 6. Adjusted school-level proportion of White students who met aerobic capacity Healthy Fitness Zone standards before (2014/15) and during PE Works (2015/16 – 2018/19) under observed and predicted PE Works conditions**


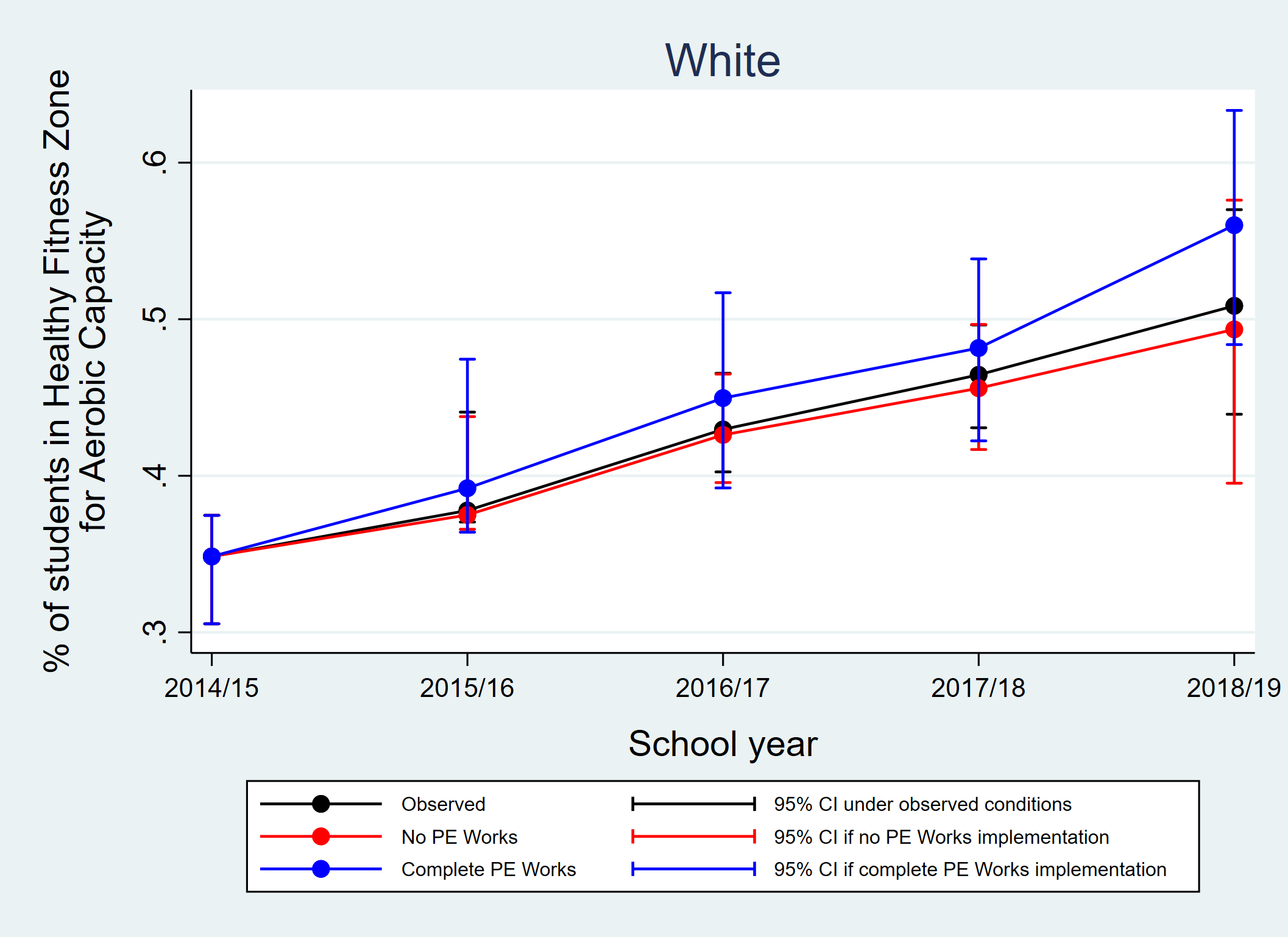


**Supplementary Figure 7. Adjusted school-level proportion of free or reduced-price meal (FRPM) eligible students who met aerobic capacity Healthy Fitness Zone standards before (2014/15) and during PE Works (2015/16 – 2018/19) under observed and predicted PE Works conditions**


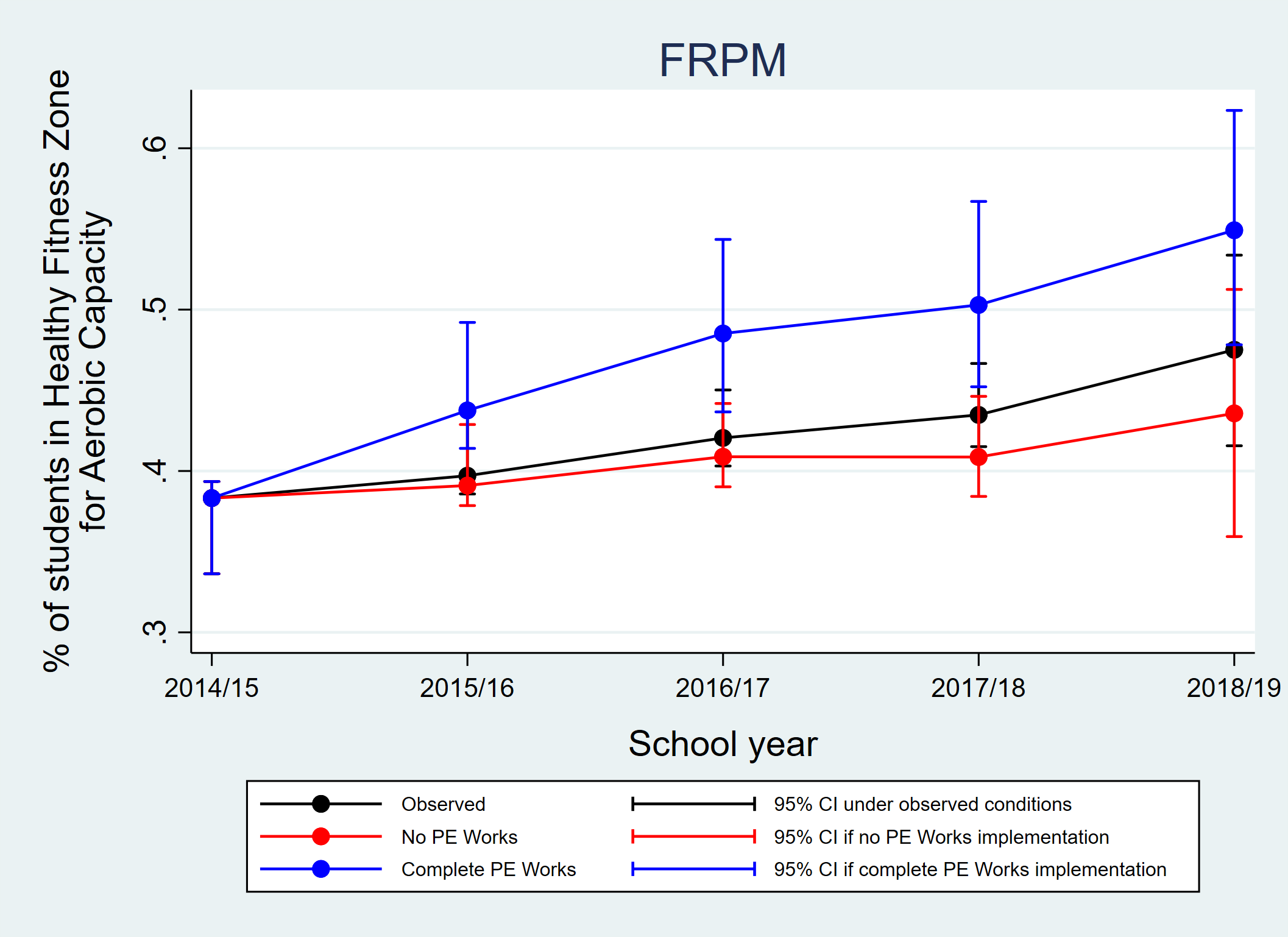


**Supplementary Figure 8. Adjusted school-level proportion of non-free or reduced-price meal (FRPM) eligible students who met aerobic capacity Healthy Fitness Zone standards before (2014/15) and during PE Works (2015/16 – 2018/19) under observed and predicted PE Works conditions**


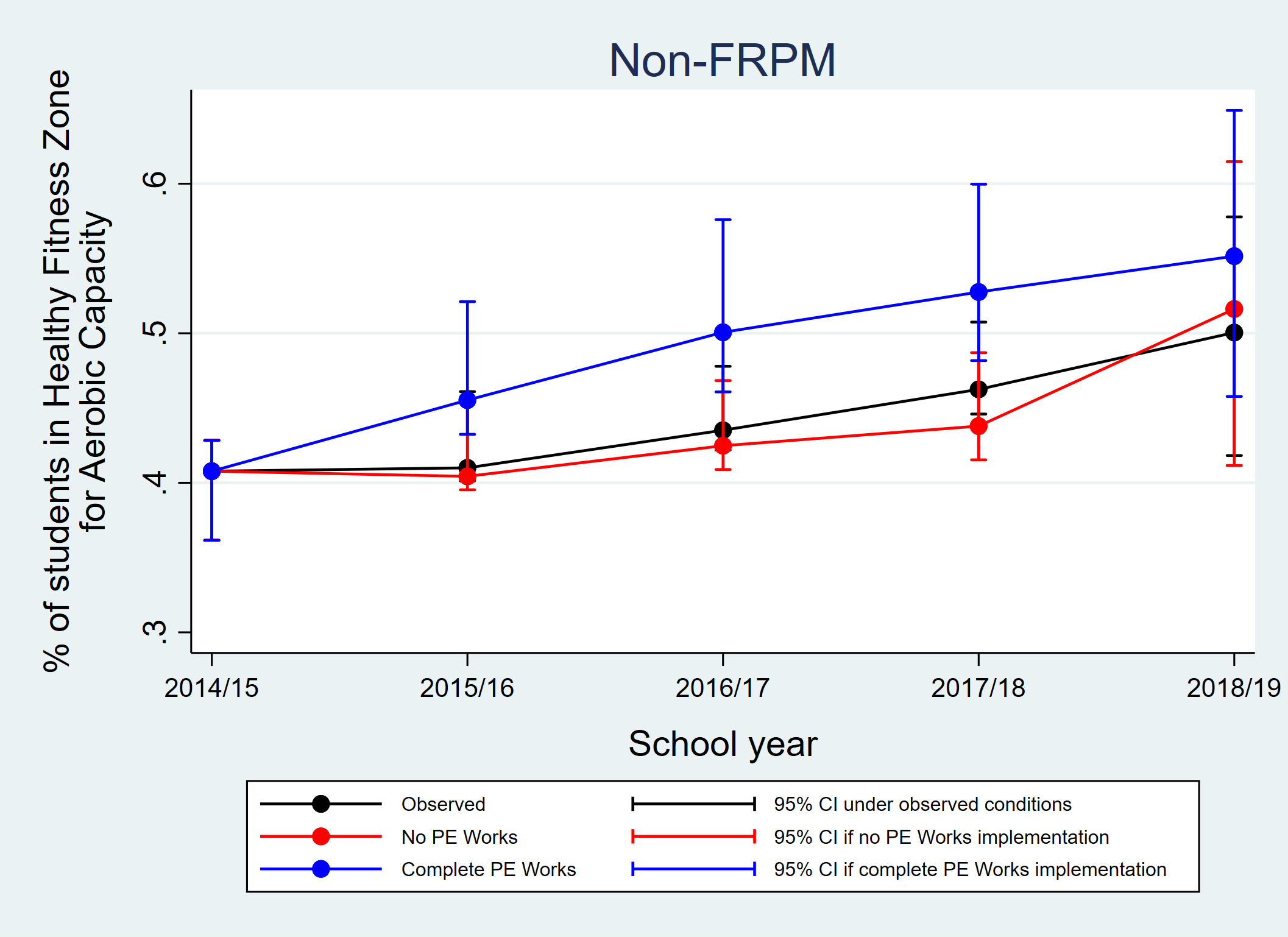

Supplement: Supplementary file 1 — Supplementary Material 1 [file 12889_2024_20673_MOESM1_ESM.docx]
